# Pardaxin Promoted Differentiation and Maturation of Leukemic Cells via Regulating TLR2/MyD88 Signal against Cell Proliferation

**DOI:** 10.1155/2019/7035087

**Published:** 2019-02-24

**Authors:** Wu-Ching Uen, Chen-Yen Choong, Chen-Jei Tai, Cheng-Jeng Tai

**Affiliations:** ^1^Department of Hematology and Oncology, Shin Kong Wu Ho-Su Memorial Hospital, Taipei, Taiwan; ^2^School of Medicine, Fujen Catholic University, New Taipei City, Taiwan; ^3^Department of Traditional Chinese Medicine, Department of Internal Medicine, Taipei Medical University Hospital, Taipei, Taiwan; ^4^Department of Obstetrics and Gynecology, School of Medicine, College of Medicine, Taipei Medical University, Taipei, Taiwan; ^5^Traditional Herbal Medicine Research Center, Taipei Medical University Hospital, Taipei, Taiwan; ^6^Division of Hematology and Oncology, Department of Internal Medicine, Taipei Medical University Hospital, Taipei, Taiwan; ^7^Division of Hematology and Oncology, Department of Internal Medicine, School of Medical, College of Medicine, Taipei Medical University, Taipei, Taiwan

## Abstract

**Objective:**

Leukemia is a cancer of the blood cells. Leukemic THP-1 and U937 cells were used in this study as monocytic effectors cells for proliferation responses and macrophage-like cells induction in leukemia. Pardaxin is an antimicrobial peptide isolated from the marine fish species.

**Methods:**

After treatment for 5 days, pardaxin significantly suppressed cell viability and arrested cell cycle at G0/G1 phase in leukemic cells which were evaluated.

**Results:**

Pardaxin also induced cell differentiation and maturation of THP-1 and U937 cells into macrophage-like cells with phagocytotic ability. Moreover, pardaxin elevated the expression of MyD88 but not toll-like receptor (TLR)-2 in both leukemic cells. TLR-2 blocking peptide was used to confirm that pardaxin attenuated phagocytotic ability and superoxide anion production in leukemic cells via activating MyD88 protein.

**Conclusions:**

These findings suggested that pardaxin has a therapeutic potential for leukemia.

## 1. Introduction

Antimicrobial peptides (AMPs) have been known to belong to a huge family of peptide molecules that typically contain less than 100 amino acids and they exist in various types of cells in vertebrates and invertebrates. Previous studies have reported that AMPs facilitate human health and reduce the cancer risk [1]. AMPs play crucial roles in innate system, angiogenesis, and anticancer processes [2–4], which specifically target certain proteins on the membrane of cancer cells and induce cell death, thus exhibiting potent toxicity in targeted cancer cells. Therefore, they have the potential to be applied on antitumor therapy [5, 6]. The present study investigates the anticancer role of an AMP pardaxin in leukemic cell lines along with its potential molecular mechanism. Pardaxin (GFFALIPKIISSPLFKTLLSAVGSALSSSGGQE) is an antimicrobial peptide (AMP) with 33-amino-acids, which is isolated from the marine fish species. Pardaxin shows antibacterial activities and inhibits various cancer cells including canine perianal gland adenomas [7], bladder-associated tumors [8], human fibrosarcoma cells [4], murine fibrosarcoma cells [9], and buccal pouch carcinogenesis [10].

Leukemia is the most common hematological malignancy. Current therapeutic options include chemotherapy, differentiation inducers, and stem cell transplantation. Among these, the strategy of differentiation induction is less toxic and safer than other methods [11, 12]. Additionally, several polysaccharides isolated from edible materials have been reported to stimulate cytokines production and differentiation of leukemic cells. For example,* Cordyceps sinensis* inhibited proliferation and induced differentiation in leukemic human U937 cells [13], and* Ganoderma lucidum-*derived polysaccharides could induce differentiation of human leukemic THP-1 cells [10]. A line of evidence shows that activators isolated from black soybean [14] and* Poria coco*s [15] exert antileukemic activity via inducing differentiation in U937 and HL-60 leukemic cells. Moreover, an AMPs epinecidin-1 has been found to regulate the toll-like receptor (TLR)-2/MyD88 pathway in RAW264.7 macrophage [16] and exert anticancer activity for leukemic U937 cells [17]. Another AMP, nisin, obtained from lactic acid bacteria has also been reported to suppress leukemic HL-60 cells [18]. However, the effects of pardaxin on regulating human leukemic cells still remained unknown. Leukemia was classified into acute lymphoblastic leukemia (ALL) and acute myeloid leukemia (AML). ALL is a cancer of the lymphoid line of blood cells characterized by the development of large numbers of immature lymphocytes; AML is a cancer of the myeloid line of blood cells, characterized by the rapid growth of abnormal cells that build up in the bone marrow and blood and interfere with normal blood cells. Moreover, THP-1 is a human acute monocytic leukemia cell line, and U937 is a human myeloid cell line. In this study, we evaluated the effects of pardaxin on cell proliferation and cell differentiation in human leukemic THP-1 and U937 cells.

## 2. Materials and Methods

### 2.1. Materials

TLR-2 block peptide was purchased from Novus Biologicals Inc. (Littleton, CO, USA). Trypan blue, RPMI 1640 medium, L-glutamine, and sodium pyruvate were purchased from GIBCO (Grand Island, NY, USA). Polymyxin B, nitroblue tetrazolium (NBT), propidium iodide (PI), sodium dodecyl sulfate (SDS), and dimethyl sulfoxide (DMSO) were from Sigma Chemical Co. (St. Louis, MO, USA). Fetal bovine serum (FBS) was from Hyclone (Logan, UT, USA). Human CD11b antibody was purchased from BD Biosciences PharMingen, San Diego, CA, USA. The TLR-2 antibody and MyD88 antibody were purchased from Santa Cruz Biotechnology Inc. (Burlingame, CA, USA).

### 2.2. Cell Culture

Human THP-1 cells and U937 leukemic cells were purchased from Bioresource Collection and Research Center (Hsinchu, Taiwan) and were incubated in RPMI 1640 medium supplemented with 10% fetal bovine serum, 2 mM _L_-glutamine, 1 mM sodium pyruvate, and antibiotics (100U/mL penicillin and 100 *μ*g/mL streptomycin). For assays of proliferation and differentiation, THP-1 cells (1.5 × 10^5^/mL) were treated with pardaxin dissolved in RPMI 1640 medium for 5 days. The numbers of viable cells were counted by trypan blue dye exclusion after treatment for 1, 3, and 5 days in the direct differentiation induction models [11].

### 2.3. Assay for Phagocytosis

After 5 days of differentiation induction with pardaxin, the phagocytic activity was assayed [11]. The cells were washed with phosphate-buffered saline and resuspended in medium (8 × 10^5^/mL) at day 5. Subsequently, cells were incubated with yeast (1.0× 10^6^/mL) at 37°C for 30 minutes. Finally, cells were placed on a glass slide and observed to determine phagocytic activity. Phagocytosis (as a percentage) was quantified by counting the percentage of yeast-containing cells from 200 cells.

### 2.4. Assay for Superoxide Anion Level

The level of reactive oxygen species was assayed with NBT. In brief, cells (2 × 10^5^/mL) were treated with pardaxin for 5 days, and then cells were collected and resuspended in medium (8 × 10^5^/mL). The cells were then incubated with NBT solution containing 1 mM phorbol myristate acetate at 37°C for 2 hours. NBT is reduced by reactive oxygen species to form blue-black formazan, which was dissolved with dimethyl sulfoxide, and the absorbance value was measured at 570 nm [19, 20]. The level of superoxide anion generation was compared to that of the normal group.

### 2.5. Assay for Specific Antigen Markers

The specific differentiation marker CD11b in cells was determined by flow cytometric analysis as previously described [11]. In brief, cells (1.0 × 10^5^/mL) were treated with pardaxin for 5 days, and then cells were washed with phosphate-buffered saline and incubated with CD11b antibody for 1 hour. Redundant antibody was removed by washing with phosphate-buffered saline. The expression of CD11b was analyzed by the flow cytometry.

### 2.6. Western Blot

Cells were lysed in ice-cold lysis buffer containing 20 mM Tris-HCl (pH 7.4), 1% Triton X-100, 0.1% SDS, 2 mM ethylenediaminetetraacetic acid, 10 mM NaF, 1 mM phenylmethylsulfonyl fluoride, 500 mM sodium vanadate, and 10 mg/mL aprotinin overnight. Then, the cell extract was centrifuged (12,000xg for 10 min) to recover the supernatant. The supernatant was taken as the cell extract. The cell protein was resolved on 10% sodium dodecyl sulfate-polyacrylamide gel electrophoresis (SDS-PAGE) gel and transferred to polyvinyldiene fluoride membrane. The membranes were blocked with 5% nonfat dry milk solution for 1 h and incubated overnight with primary antibodies for 4 h; subsequently, the membrane was washed 3 times each for 5 min in phosphate-buffered saline with Tween 20, shaken in a solution of horseradish peroxidase (HRP)-linked secondary antibody for 1 h, and washed 3 more times each for 5 min in PBST. The expressions of proteins were detected by enhanced chemiluminescent reagent (Millipore, Billerica, MA).

### 2.7. Statistical Analysis

Experimental results were analyzed in triplicate and expressed as means ± standard deviation (SD). The results were subjected to one-way analysis of variance (ANOVA) and Duncan's multiple range tests and the significance of differences between sample means was calculated. The* P *value < 0.05 was considered significant difference.

## 3. Results

### 3.1. The Effect of Pardaxin on Cell Survival in Leukemic Cells

**Figure 1:**
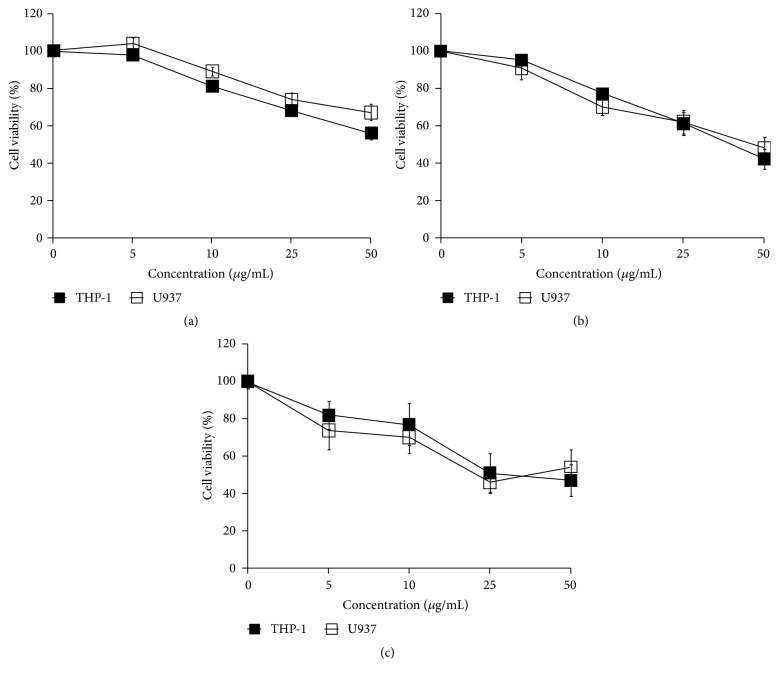
The inhibition of pardaxin on proliferation of THP-1 and U937 leukemic cells after treatment for (a) 1 day, (b) 3 days, and (c) 5 days. Result of blank (0 *μ*g/mL) group was used to normalization to other groups in days 1, 3, and 5, respectively. And the cell survival was assayed by trypan blue stain. Results were shown as mean ± SD (n = 3).

**Figure 2:**
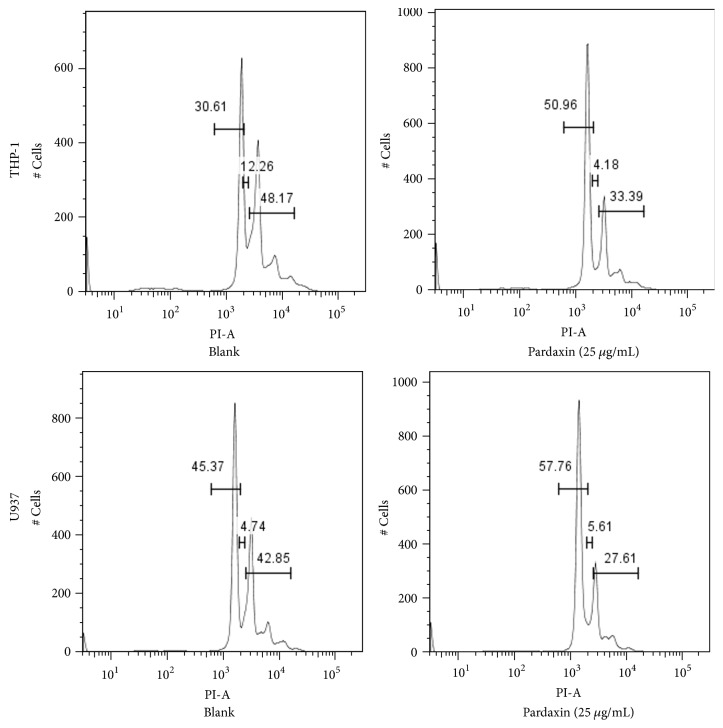
The effects of pardaxin (25 *μ*g/mL) on cell cycle of THP-1 and U937 leukemic cells were assayed by flow cytometeric analysis after treatment for 5 days. The statistical results were shown in Table 1.

Cell viability was decreased in 5, 10, 25, or 50 *μ*g/mL pardaxin-treated THP-1 and U937 leukemic cells for 1, 3, and 5 days, and there were no significant differences in pardaxin-treated groups between THP-1 and U937 leukemic cells whether at day 1, day 3, or day 5. These results indicated that pardaxin has the potential to be antileukemic (Figure 1). To understand whether other mechanisms may be involved in the inhibition of pardaxin on leukemic cells, the effect of pardaxin on cell cycle distribution in THP-1 and U937 leukemic cells was evaluated. As shown in Figure 2 and Table 1, the cell cycle was arrested in G0/G1 phase after treatment with 25 *μ*g/mL of pardaxin for 5 days in both THP-1 and U937 leukemic cells, suggesting that pardaxin treatment limited the cell proliferation of leukemic cells.

### 3.2. The Induction of Pardaxin on Cell Differentiation in Leukemic Cells

Cell differentiation was found in leukemic THP-1 cells while the cell cycle was arrested in G0 phase [11]. Specific cell markers related to macrophage differentiation were determined and showed in Table 2. After 5-day treatment, the expressions of CD11b were significantly increased by pardaxin in leukemic THP-1 cells and U937 cells from 26.8% and 45.4% to 44.3% and 55.9%, respectively.

### 3.3. The Activation of Leukemic Cells Treated by Pardaxin

**Figure 3:**
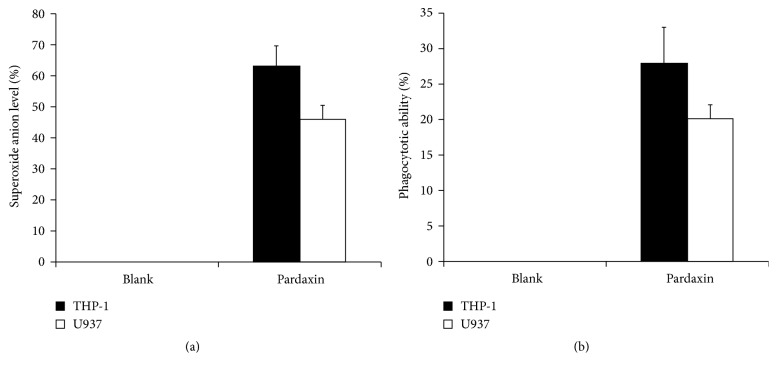
The induction effect of pardaxin on cell differentiation in THP-1 and U937 leukemic cells. The (a) superoxide anion level and (b) phagocytotic ability in the THP-1 and U937 leukemic cells treated by pardaxin for 5 days were assayed by NBT stain and microscopy, respectively. Results were shown as mean ± SD (n = 3).

Cell maturation and activity of THP-1 and U937 leukemic cells were evaluated in this study, including superoxide anion production and phagocytosis. CD11b is a marker for cell maturation of leukemic cells. As shown in Figure 3, pardaxin treatment promoted cell maturation. Also, superoxide anion level and phagocytotic ability were elevated in both THP-1 and U937 leukemic cells by treatment with pardaxin for 5 days.

### 3.4. Pardaxin Promoted THP-1 and U937 Leukemic Cells Differentiation via Regulating TLR-2/MyD88 Signals

**Figure 4:**
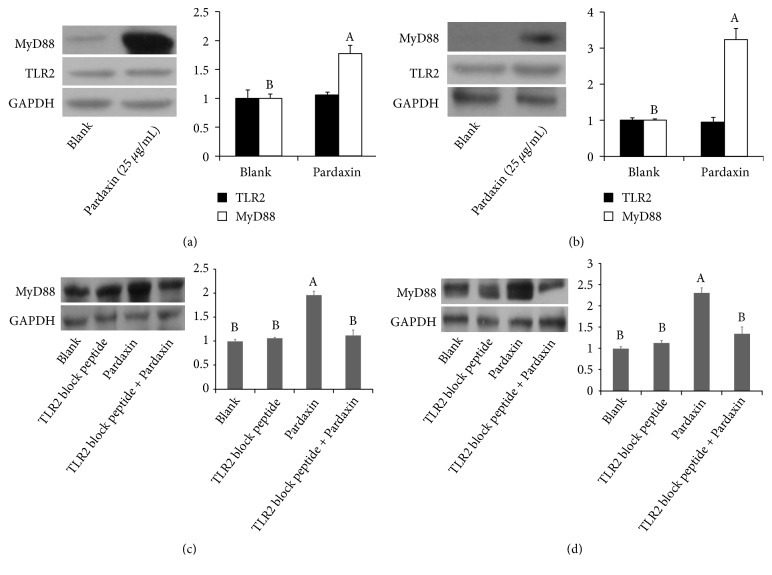
The TLR2 and MyD88 expression in (a) THP-1 and (b) U937 leukemic cells treated by pardaxin for 5 days. The attenuation of MyD88 expression treated by TLR2 block peptide in (c) THP-1 and (d) U937 leukemic cells for 5 days. Results were shown as mean ± SD (n = 3). The significant difference was shown by various letters (p<0.05).

**Figure 5:**
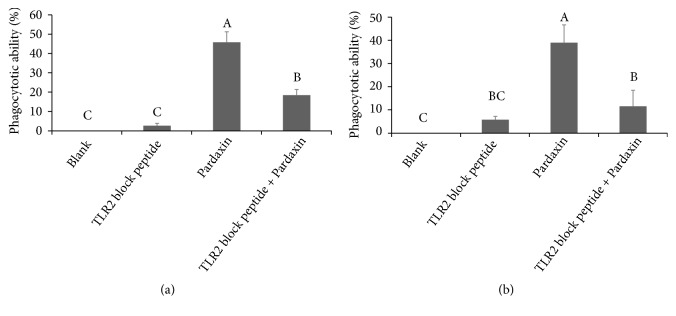
The attenuation of phagocytotic ability treated by TLR2 block peptide in (a) THP-1 and (b) U937 leukemic cells for 5 days. Results were shown as mean ± SD (n = 3). The significant difference was shown by various letters (p<0.05).

**Figure 6:**
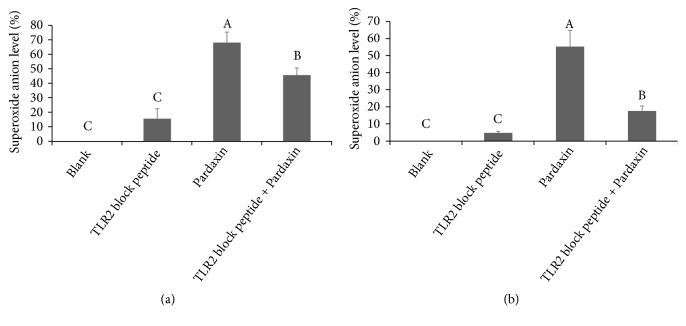
The attenuation of superoxide anion production treated by TLR2 block peptide in (a) THP-1 and (b) U937 leukemic cells for 5 days. Results were shown as mean ± SD (n = 3). The significant difference was shown by various letters (p<0.05).

**Figure 7:**
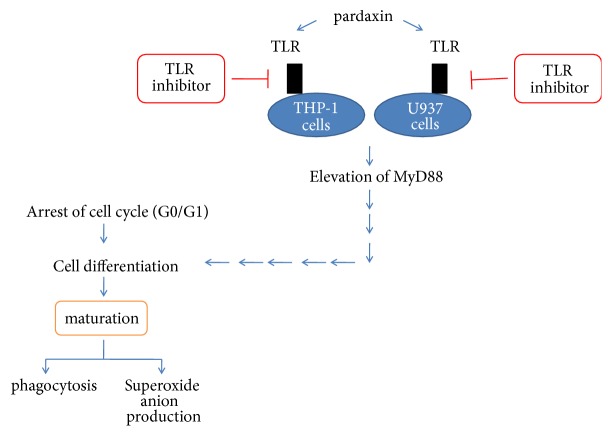
Graphical abstract for pardaxin suppressed leukemic cells.

Toll-like receptor (TLR)-2 has an important role in the activation and maturation of leukemic cells, and MyD88 protein is a downstream molecule in the TLR pathway. The expression of MyD88 was markedly increased after treatment with pardaxin for 5 days in leukemic THP-1 and U937 cells, whereas TLR-2 level was not influenced in each group (Figure 4), revealing that pardaxin induced cell differentiation via promoting MyD88 level in leukemic THP-1 and U937 cells. In order to confirm the mechanism of pardaxin on TLR-2/MyD88 pathway and leukemic cell maturation, TLR-2 block peptide was used to suppress TLR-2 activation and MyD88 expression, and the results demonstrated that pardaxin treatment for 5 days significantly increased MyD88 level in leukemic THP-1 and U937 cells, but this effect was abolished by treatment with TLR-2 blocking peptide (Figure 5). Similar results were found in superoxide anion production and phagocytotic ability in leukemic THP-1 cells (Figure 6) and U937 cells (Figure 7), and the elevations of superoxide anion production and phagocytotic ability by treatment with pardaxin for 5 days were attenuated by TLR-2 blocking peptide treatment in leukemic THP-1 and U937 cells.

## 4. Discussion

To induce cell differentiation is an important therapeutic method in hematological cancers like leukemia. Tumor cell differentiation ends cancer cells' immortality, thus stopping the cell growth and proliferation. One approach for treating leukemia is differentiation therapy that outlines a treatment plan to eliminate maturation blockage and allows cell differentiation to take place [21]. Terminal differentiation induction represents an alternative approach in leukemia treatment, which would generate leukemia cells with limited replicate capacity that ultimately undergoes apoptosis [22]. Numerous compounds have been screened to treat leukemia. For example, differentiation induced all-trans retinoic acid is effective in leukemia therapy [23]. It was found that arsenic trioxide synergizes with all-trans retinoic acid to enhance terminal differentiation, and tyrosine kinase inhibitor gefitinib markedly activates all-trans retinoic acid-induced differentiation of myeloid cell lines [24–26]. Other differentiation inducers of myeloid leukemia cells have been well documented for leukemia therapy, such as 12-O-tetradecanoylphorbol-13-acetate and vitamin D3 [27, 28].

Recently, several peptides have been found to suppress leukemia cancer like THP-1 leukemic cells by inducing plasma membrane damage, including LPSBD0 and LPSBD2 peptides [29]. Epinecidin-1 peptide also has been found to regulate the toll-like receptor (TLR)-2/MyD88 pathway in RAW264.7 macrophage [16] and exert anticancer activity for leukemia U937 cells [17]. In addition to cell death, cell differentiation was also important in pardaxin-treated THP-1 and U937 cells in present study. The effect of pardaxin on cell differentiation was confirmed by CD11b marker from monocytic phenotypes to macrophage-like cells and the elevated cell adherence percentage. According to phagocytotic ability assay and cell cycle analysis, we suggest that pardaxin promotes the activation and maturation of macrophage-like cells. We found that superoxide anion production and phagocytotic ability were markedly increased and cell cycle was arrested at G0/G1 phase after treating pardaxin for 5 days.

Acute myeloid leukemia (AML) is the most common acute leukemia in adults, which is a heterogeneous disorder of hematopoietic progenitor cells. AML arise from the acquisition and accumulation of genetic and/or epigenetic lesions by progenitor hematopoietic cells, resulting in disruption of differentiation and increased proliferation and leading to blast-cell accumulation in the bone marrow/other tissues. At present, the approach for AML therapy is chemotherapy by using a combination of cytotoxic agents that kill highly proliferating cells. There are no changes in the treatment of AML in these years; it is necessary to develop novel agents for improving cure rates and decreasing the toxicity [30]. Differentiation therapy may be a very interesting possibility that allows more efficacious and less toxic therapeutic regimen. The role of TLRs on mature myeloid cells in triggering innate immune response as well as the subsequent development of adaptive immune response to microbial pathogens is well established. However, different reports have shown that functional TLRs are also expressed on hematopoietic stem and progenitor cells and therefore play a role in hematopoiesis during infection [31]. Different TLR agonists show antitumor effects by different mechanisms: activating immune responses to suppress growth of tumors; increasing dendritic cell maturation and presentation; activating NK or T cytotoxic cells or increasing infiltrating immune cells in the tumors. Nevertheless, we hypothesize that pardaxin may have a direct effect on the leukemic cells in addition to their known immune activating effect.

## 5. Conclusion

Our findings suggest that pardaxin induced selecting leukemic cells differentiation into macrophage-like cells with immunostimulatory function, including phagocytotic ability and superoxide anion production. The possible clinical impact of using this peptide in patients with monocytic leukemia will need further investigation.

## Figures and Tables

**Table 1:** The effect of pardaxin on cell cycle in THP-1 and U937 leukemic cells after treatment for 5 days.

Cell cycle (%)	THP-1		
	G0/G1	S	G2/M
Blank	30.61±2.16_ _^b^	13.41±0.89_ _^a^	49.46±1.58_ _^a^
Pardaxin (25 *μ*g/mL)	50.96±1.65_ _^a^	4.32±0.67_ _^b^	35.62±1.13_ _^b^

	U937		

Blank	44.62±1.13_ _^b^	4.91±0.78	45.79±1.77_ _^a^
Pardaxin (25 *μ*g/mL)	57.76±1.29_ _^a^	5.85±1.08	29.66±1.43_ _^b^

Results were shown as mean ± SD (n = 3). The significant difference was shown by various letters between blank and pardaxin treatment group (p<0.05).

**Table 2:** The effect of pardaxin on mature CD marker (CD11b) in THP-1 and U937 leukemic cells after treatment for 5 days.

CD11b (%)	THP-1	U937
Blank	26.8±1.2_ _^b^	45.4±0.8_ _^b^
Pardaxin (25 g/mL)	44.3±2.2_ _^a^	55.9±1.4_ _^a^

Results were shown as mean ± SD (n = 3). The significant difference was shown by various letters between blank and pardaxin treatment group (p<0.05).

## Data Availability

The data used to support the findings of this study are available from the corresponding author upon request.
